# Hyperinsulinemia augments endothelin‐1 protein expression and impairs vasodilation of human skeletal muscle arterioles

**DOI:** 10.14814/phy2.12895

**Published:** 2016-08-22

**Authors:** Abeer M. Mahmoud, Mary R. Szczurek, Brian K. Blackburn, Jacob T. Mey, Zhenlong Chen, Austin T. Robinson, Jing‐Tan Bian, Terry G. Unterman, Richard D. Minshall, Michael D. Brown, John P. Kirwan, Shane A. Phillips, Jacob M. Haus

**Affiliations:** ^1^ Department of Kinesiology and NutritionUniversity of Illinois at ChicagoChicagoIllinois; ^2^ Integrative Physiology LaboratoryUniversity of Illinois at ChicagoChicagoIllinois; ^3^ Department of Physical TherapyUniversity of Illinois at ChicagoChicagoIllinois; ^4^ Department of Pharmacology and AnesthesiologyUniversity of Illinois at ChicagoChicagoIllinois; ^5^ Department of MedicineDivision of Endocrinology, Diabetes and MetabolismUniversity of Illinois at ChicagoChicagoIllinois; ^6^ Department of PathobiologyLerner Research InstituteCleveland ClinicClevelandOhio

**Keywords:** Endothelin‐1, hyperinsulinemia, microvasculature, nitric oxide, skeletal muscle

## Abstract

Hyperinsulinemia is a hallmark of insulin resistance‐associated metabolic disorders. Under physiological conditions, insulin maintains a balance between nitric oxide (NO) and, the potent vasoconstrictor, endothelin‐1 (ET‐1). We tested the hypothesis that acute hyperinsulinemia will preferentially augment ET‐1 protein expression, disrupt the equilibrium between ET‐1 expression and endothelial NO synthase (eNOS) activation, and subsequently impair flow‐induced dilation (FID) in human skeletal muscle arterioles. Skeletal muscle biopsies were performed on 18 lean, healthy controls (LHCs) and 9 older, obese, type 2 diabetics (T2DM) before and during (120 min) a 40 mU/m^2^/min hyperinsulinemic‐euglycemic (5 mmol/L) clamp. Skeletal muscle protein was analyzed for ET‐1, eNOS, phosphorylated eNOS (p‐eNOS), and ET‐1 receptor type A (ETAR) and B (ETBR) expression. In a subset of T2DM (*n *=* *6) and LHCs (*n* = 5), FID of isolated skeletal muscle arterioles was measured. Experimental hyperinsulinemia impaired FID (% of dilation at ∆60 pressure gradient) in LHCs (basal: 74.2 ± 2.0; insulin: 57.2 ± 3.3, *P* = 0.003) and T2DM (basal: 62.1 ± 3.6; insulin: 48.9 ± 3.6, *P* = 0.01). Hyperinsulinemia increased ET‐1 protein expression in LHCs (0.63 ± 0.04) and T2DM (0.86 ± 0.06) compared to basal conditions (LHCs: 0.44 ± 0.05, *P* = 0.007; T2DM: 0.69 ± 0.06, *P* = 0.02). Insulin decreased p‐eNOS (serine 1177) only in T2DM (basal: 0.28 ± 0.07; insulin: 0.17 ± 0.04, *P* = 0.03). In LHCs, hyperinsulinemia disturbed the balance between ETAR and ETBR receptors known to mediate vasoconstrictor and vasodilator actions of ET‐1, respectively. Moreover, hyperinsulinemia markedly impaired plasma NO concentration in both LHCs and T2DM. These data suggest that hyperinsulinemia disturbs the vasomotor balance in human skeletal muscle favoring vasoconstrictive pathways, eventually impairing arteriolar vasodilation.

## Introduction

Compensatory hyperinsulinemia associated with insulin resistance is considered one of the primary mechanisms that promotes vascular dysfunction and the subsequent development of cardiovascular disease (CVD) (Muniyappa et al. 2007). Understanding the mechanism by which hyperinsulinemia induces vascular dysfunction is essential for advancing treatment and prevention strategies of insulin resistance‐related vascular complications. At physiological levels, insulin regulates vascular function by maintaining the balance between endothelial‐derived nitric oxide (NO) and, the potent vasoconstrictor, endothelin‐1 (ET‐1). Insulin initiates signaling cascades through both phosphatidylinositol 3‐kinase (PI3K) and mitogen‐activated protein kinase (MAPK) pathways that subsequently activate eNOS phosphorylation and ET‐1 protein expression, respectively (Muniyappa et al. 2007). The concept of selective insulin resistance, characterized by impairment of PI3K signaling with preservation of MAPK signaling, may be the mechanism by which chronic hyperinsulinemia promotes vascular dysfunction (Kim et al. 2006). Thus, the unopposed activation of the MAPK signaling arm of insulin action, and subsequent transcriptional upregulation of ET‐1, will favor the vasoconstrictor effects of insulin. However, it remains unclear whether or not mere hyperinsulinemia, in isolation from insulin resistance, augments ET‐1 signaling and, thus, disturbing the vasomotor balance and compromising endothelial function.

Numerous studies have reported enhanced limb blood flow in response to insulin stimulation (Clerk et al. 2006; Fujita et al. 2006; Eggleston et al. 2007), whereas others demonstrate either null effect or reduction in blood flow in lean, healthy individuals (Hedman et al. 2001; Arcaro et al. 2002; Morgantini et al. 2012). Insulin infusion in rats has been shown to increase plasma ET‐1 and precipitate hypertension (Juan et al. 2004; Potenza et al. 2005). Similarly, in vitro studies have consistently demonstrated increased ET‐1 gene expression in insulin‐treated endothelial cells (Formoso et al. 2006; Yang and Li 2008). Collectively, these observations support the notion that hyperinsulinemia, not confounded by insulin resistance, induces ET‐1 production and disturbs vasomotor balance.

The effect of insulin infusion on ET‐1 signaling in humans has mainly been deduced from circulating concentrations of ET‐1 (Ferri et al. 1995b,c; Seljeflot et al. 1998; Surdacki et al. 1999). However, circulating ET‐1 concentrations do not correlate with vasoconstrictor activity in peripheral tissues as ET‐1 acts predominantly in a local fashion after being released from endothelial cells (Wagner et al. 1992). This emphasizes the importance of measuring ET‐1 and microvascular responses to insulin in peripheral tissues. Among the insulin sensitive tissues, skeletal muscle accounts for more than 80% of insulin‐stimulated glucose disposal (Defronzo et al. 1985) and receives more than 25% of the cardiac output at rest (Korthuis 2011). Also, skeletal muscle insulin resistance is the primary defect in the development of T2DM and future risk of CVD (Petersen et al. 2007). To our knowledge, no data exist that examine human isolated skeletal muscle microvessel function in response to experimental hyperinsulinemia. Furthermore, direct comparisons between a healthy reference group and T2DM subjects are also lacking. Thus, the primary objective of this study was to investigate the effect of systemic hyperinsulinemia on human skeletal muscle microvascular function and differentiate the potential differences in chronic hyperinsulinemia, as experienced with insulin resistance and T2DM versus acute experimental hyperinsulinemia in lean healthy adults. We hypothesized that acute hyperinsulinemia would disturb the balance of vasoactive mediators through biased augmentation of the ET‐1 pathway, with subsequent impairment of endothelial function. In healthy and T2DM subjects, we measured skeletal muscle protein expression of the major vasoactive mediators (ET‐1 and eNOS) collected under basal and insulin‐stimulated conditions of a hyperinsulinemic‐euglycemic clamp. In addition, microvessels isolated from skeletal muscle biopsy samples, obtained at basal and insulin‐stimulated conditions, were studied via in vitro flow‐induced dilation (FID).

## Materials and Methods

### Study design

We designed this cross‐sectional study to obtain as much information as possible about skeletal muscle microvessel responses to experimental hyperinsulinemia. Our intent was to examine these responses in a population of obese, hypertensive, T2DM subjects and directly contrast these findings with lean healthy controls (LHCs). Comparisons to a LHC group, representing an implied state of good health, allowed for a reference of “normal” protein expression and physiological responses, thus highlighting the importance of the disease phenotype. Hyperinsulinemic‐euglycemic clamps were used to induce experimental hyperinsulinemia and characterize whole‐body insulin sensitivity. Skeletal muscle needle biopsies were obtained during basal and insulin‐stimulated conditions of the clamp procedure for each subject. Skeletal muscle tissue samples were probed for protein expression of vasoactive regulatory proteins, and fresh tissue samples (when available) were dedicated to the isolation, and ex vivo study, of resistance microvessels. To our knowledge, this is one of the first studies of isolated skeletal muscle resistance arterioles from humans due to tissue limitations and technical burden. Despite these technical challenges, we were successful in obtaining FID data from both LHCs and T2DM participants. Also, skeletal muscle tissue and plasma samples, when available, were used for histological analysis of capillary density and NO bioavailability, respectively.

### Subjects

Participants were recruited from the Cleveland, OH, and Chicago, IL, metropolitan areas. All subjects were screened via health history, medical exam, resting EKG, and fasting blood chemistry in the Clinical Research Centers of the Cleveland Clinic and the University of Illinois at Chicago. Glucose tolerance was characterized by a 75 g oral glucose tolerance test (OGTT) following a standard fasting period for all subjects. Individuals were excluded if they used nicotine, had undergone greater than 2‐kg weight change in the last 6 months, or had evidence of hematological, renal, hepatic, or CVD. LHCs were excluded if results of OGTT indicated impaired fasting glucose or impaired glucose tolerance. T2DM subjects self‐reported diabetes duration of 4 ± 1 years. All studies were approved by the Institutional Review Boards of the Cleveland Clinic and the University of Illinois at Chicago and performed in accordance with the Declaration of Helsinki. Written informed consent was obtained from all research participants during the initial screening visit.

### Metabolic control

All subjects were instructed to maintain their regular dietary eating habits and completed 3‐day diet records (2 weekday and 1 weekend day). Subjects were asked to refrain from physical activity outside of their normal activities of daily living, consumption of alcoholic beverages, and consumption of foods and beverages containing caffeine for 48 h prior to the metabolic testing day. On the day preceding metabolic testing, subjects were counseled to consume ~55% of calories as carbohydrate in order to meet a goal of 250 g (Solomon et al. 2011; Haus et al. 2013). The evening prior to testing, participants were provided a balanced metabolic meal (55% carbohydrate, 35% fat, and 10% protein). The meal composition was free of foods that are known to contain high amounts of nitrates. After the meal consumption, the participants fasted overnight for a period of 10–12 h. All participants were asked to withhold medications and supplements the morning of metabolic testing that were known to influence primary outcome variables. This included all medications for the treatment of allergies, asthma, hypercholesterolemia, hypertension, pain, and T2DM. A full list of medication use is provided in Table 1. Female participants (*n* = 13), who were premenopausal with regular menstrual cycles, were studied in the follicular phase. Two women were postmenopausal without use of hormone replacement therapy.

**Table 1:** Medication use

Drug class	LHC (*n*)	T2DM (*n*)
Aldosterone receptor antagonist	0	1
Alpha blocker	0	2
Angiotensin converting enzyme inhibitor	0	1
Angiotensin II receptor antagonists	0	2
Antiallergy	4	3
Antimineralcorticoid	1	1
Beta‐blocker	0	2
Bronchodilator	1	1
Calcium channel blocker	0	1
Diuretic	0	2
DPP4 inhibitor	0	2
Insulin	0	3
Metformin	0	5
NSAID	2	4
Oral contraceptive	3	0
Proton pump inhibitor	1	1
Statin	0	4
Sulfonylurea	0	3

LHC, lean healthy control.

### Insulin sensitivity

Whole‐body insulin sensitivity was assessed using the hyperinsulinemic (40 mU/m^2^/min)‐euglycemic (5 mmol/L) clamp procedure. Arterialized venous blood was sampled for glucose concentrations at 5‐min intervals and continued over the course of 2 h (120 min) (YSI 2300; STAT Plus, Yellow Springs, OH). Adjustments to the glucose infusion rate were made according to the calculations of Defronzo et al. (1979). Mean space‐corrected exogenous glucose infusion rates during the final 30 min of steady‐state hyperinsulinemia are presented as peripheral tissue glucose disposal rates (GDR: mg/kg/min). We have previously described these procedures in detail (Haus et al. 2009; Solomon et al. 2010, 2013; Williamson et al. 2015).

### Skeletal muscle biopsy

Skeletal muscle needle biopsies (*vastus lateralis*) were performed during the baseline (0 min) and insulin‐stimulated (120 min) periods of the hyperinsulinemic‐euglycemic clamp procedure (Haus et al. 2007; Williamson et al. 2015). Infusion of steady‐state insulin and glucose was not terminated until completion of the muscle biopsy procedure. Muscle tissue was immediately blotted, trimmed of adipose and connective tissue, and aliquoted as described below. Skeletal muscle tissue was either: (1) flash frozen in liquid nitrogen for protein expression analyses; (2) placed in ice‐cold (4°C) HEPES buffer solution (pH 7.4) for resistance vessel isolation and subsequent FID experiments; and/or (3) mounted in tragacanth gum and frozen in liquid nitrogen‐cooled isopentane for histological analysis. Protein and histology samples were stored at −80°C until analysis. FID experiments were completed immediately following biopsy.

### FID experiments

Resistance arterioles of ~50 *μ*m in diameter and ~2 mm in length were carefully dissected from the skeletal muscle tissue and cleaned of fat and connective tissue. In an organ perfusion chamber, single vessels were cannulated with glass micropipettes (outer tip diameter ∼40 *μ*m) filled with cold bicarbonate buffer consisting of 123 mmol/L NaCl, 4.4 mmol/L KCl, 2.5 mmol/L CaCl_2_, 1.2 mmol/L MgSO_4_, 20 mmol/L NaHCO_3_, 1.2 mmol/L KH_2_PO_4_, and 11 mmol/L glucose. Both ends of the vessel were secured with 10‐0 nylon Ethilon monofilament suture, and the vessels were maintained at an intraluminal pressure of 20 mmHg for 30 min. Each preparation was transferred to the stage of an inverted microscope (magnification ×200) attached to a video camera, monitor, and video‐measuring device (model VIA‐100; Boeckeler Instruments, Tucson, AZ). The external bathing medium was continuously superfused with heated buffer solution (pH = 7.4 ± 0.05, Po_2_ = 140 ± 10 mmHg) aerated with a gas mixture of 21% O_2_–5% CO_2_–74% N_2_ and maintained at 37°C. The pressure was slowly increased to 100 mmHg and maintained for 30 min. Vessels were preconstricted 30–50% with ET‐1 (100–200 pM). Vessels that did not constrict >30% were excluded from the analysis. Flow was produced by simultaneously changing the heights of the reservoirs in equal and opposite directions to generate an intraluminal pressure gradient of ∆10–∆100 cmH_2_O (equivalent to ~7–70 mmHg), which covers the physiological range of arteriolar pressure in the human body (Phillips et al. 2007; Grizelj et al. 2015). In separate experiments, vasoreactivity measures were determined in response to incremental doses of acetylcholine (ACh) (10^−9^–10^−4^ mol/L). Steady‐state internal arterial diameters were measured before and during intraluminal flow or ACh in the absence or presence of the NO synthase (NOS) inhibitor N^*ω*^‐nitro‐l‐arginine methyl ester (L‐NAME; 10^−4^ mol/L). Maximal diameter of every vessel was determined in the presence of papaverine (10^−4^ mol/L), and the diameter in response to flow at a gradient of 100 cmH_2_O was measured in the presence of papaverine first in one direction, and then with the gradient reversed to verify pipette resistance matching (Phillips et al. 2007).

### Immunoblotting

Basal and insulin‐stimulated protein expression of eNOS (sc‐654; Santa Cruz Biotechnology, Dallas, TX), p‐eNOS (serine 1177) (9570S; Cell Signaling Technology, Beverly, MA), endothelin‐1 (ET‐1) (ab2786; Abcam, Cambridge, MA), endothelin‐converting enzyme (ECE) (sc‐376017; Santa Cruz Biotechnology), ETAR, and ETBR (ab178454 & ab129102; Abcam) was achieved via immunoblotting for all subjects. Of technical note, the ET‐1 antibody detects the 203 amino acid pre‐proendothelin‐1 protein using an epitope corresponding to amino acids 8–16. This epitope is shared by all endothelin‐1 posttranslational products (pre‐proendothelin‐1 (24 kDa), pro‐endothelin‐1 (4 kDa) and endothelin‐1 (2 kDa)). Given the difficulties in resolving small protein products via SDS‐PAGE, it is widely accepted for the 24 kDa pre‐proendothelin‐1 to be representative of active ET‐1.

Approximately 10–15 mg (wet weight) of frozen muscle tissue from each sample was homogenized (lysing matrix D beads; FastPrep^®^‐24 homogenizer, MP Biomedicals, Santa Ana, CA) in ice‐cold buffer consisting of 20 mmol/L Tris‐HCl (pH 7.5), 150 mmol/L NaCl, 1 mmol/L Na_2_ EDTA, 1 mmol/L EGTA, 2.5 mmol/L Na pyrophosphate, 1 mmol/L *β*‐glycerophosphate, 1 mmol/L Na_3_VO_4_, 1% Triton, and 1 *μ*g/mL leupeptin (Cell Signaling Technology) with an added protease and phosphatase inhibitor cocktail (Sigma Aldrich, St. Louis, MO). Total protein concentration was determined via BCA Protein Assay (Pierce Biotechnology, Rockford, IL), and 30 *μ*g protein were separated via 10% SDS‐PAGE and immunoblotted using primary antibodies. Protein expression of glyceraldehyde 3‐phosphate dehydrogenase (GAPDH) (D16H1; Cell Signaling Technology) or *β*‐Actin (612657; BD Biosciences, San Jose, CA) was utilized as a loading control. Densitometry was performed using NIH Image J software.

### Plasma NO

As a surrogate marker of NO bioavailability, concentrations of nitrate and nitrite, stable end products of NO metabolism, were measured in basal and insulin‐stimulated plasma samples using the Griess reaction (Cayman Chemicals, Ann Arbor, MI) (Solomon et al. 2011). Briefly, nitrate was converted into nitrite utilizing nitrate reductase; then the Griess reagents were added which convert nitrite into a dark purple azo compound. The absorbance was measured at 540 nm using a plate reader. Samples were run in triplicates, and the concentration of nitrate was calculated using a nitrate standard curve.

### Capillary density

Serial 10‐*μ*m cross‐sections were obtained using a cryostat (Leica CM 3050; Leica Microsystems, Bannockburn, IL) and mounted on poly‐ l‐lysine‐coated glass slides. Capillaries were identified using a periodic acid‐Schiff stain (Sigma) following digestion of glycogen by amylase and hematoxylin counterstain, as described previously (Solomon et al. 2011). Images were acquired by using a Nikon microscope and analyzed using NIS‐Elements Microscope Imaging Software (Nikon, Melville, NY). The number of capillaries per square millimeter of surface area and the average number of capillary contacts per fiber were determined.

### Statistical analyses

Baseline subject characteristics for each group (LHCs vs. T2DM) were compared using an independent samples *t*‐test. A two‐way (group [LHCs vs. T2DM]) × condition [basal vs. insulin]) ANOVA, with repeated measures for “condition,” was used to compare protein expression and plasma NO data. FID data collected at basal, basal + L‐NAME, insulin, insulin + L‐NAME, ACh basal, ACh basal + L‐NAME, ACh insulin, and ACh insulin + L‐NAME were compared using a two‐way (group [LHCs vs. T2DM] × dose [10, 20, 40, 60, 100 cmH_2_O]) ANOVA. Bonferroni/Dunn post hoc tests were used for multiple comparisons when appropriate. Univariate analysis of variance was used to explore absolute change (Δ; insulin – basal), percentage Δ (% Δ; insulin), and capillary density data for group differences. Bivariate correlation analyses were performed with Pearson correlation coefficients. SPSS v22 (IBM, Armonk, New York) was used to perform all statistical analyses. *P* < 0.05 was considered significant and data are presented as mean ± SEM.

## Results

### Subject characteristics

Baseline subject characteristics including anthropometric and metabolic variables are presented in Table 2 for each group. As expected, and by design, LHCs were significantly different from T2DM subjects in age, body composition, systolic blood pressure, and glucose metabolism with the exception for fasting insulin and steady‐state plasma insulin (90–120 min) during the hyperinsulinemic‐euglycemic clamp.

**Table 2:** Subject characteristics

Variable	LHC	T2DM
*n*	18 (7 ♂)	9 (5 ♂)
Age, year	31 ± 2	58 ± 4^a^
Weight, kg	62.8 ± 2.8	104.7 ± 7.7^a^
BMI, kg/m^2^	22.3 ± 0.6	34.1 ± 2.1^a^
Body fat, %	25.3 ± 1.4	40.2 ± 2.8^a^
Systolic BP, mmHg	116 ± 4	135 ± 6^a^
Diastolic BP, mmHg	69 ± 3	79 ± 4
FPG, mg/dL	91 ± 1	127 ± 12^a^
FPI, *μ*U/mL	8.4 ± 1.4	8.0 ± 1.0
HbA1c, %	5.4 ± 0.1	7.2 ± 0.6^a^
2 h‐OGTT, mg/dL	101 ± 3	233 ± 39^a^
GDR, mg/kg/min	6.6 ± 0.4	2.8 ± 0.3^a^
Clamp Insulin, *μ*U/mL	91.0 ± 10.2	89.2 ± 6.0
Total chol, mg/dL	156 ± 6	152 ± 10
HDL, mg/dL	63 ± 4	53 ± 5
LDL, mg/dL	79 ± 7	79 ± 7
VLDL, mg/dL	15 ± 1	21 ± 5
TG, mg/dL	74 ± 6	104 ± 26

Data represent mean ± SEM. a.u., arbitrary units; BMI, body mass index; chol, cholesterol; fat%, percentage of body fat; FPG, fasting plasma glucose; FPI, fasting plasma insulin; GDR; hyperinsulinemic‐euglycemic clamp‐derived glucose disposal rate; clamp insulin, steady‐state plasma insulin concentrations at end of clamp; HbA1c, hemoglobin A1c; LHC, lean healthy control; OGTT, oral glucose tolerance test; TG, triglycerides.

a Significant difference between LHC and T2DM (*P* < 0.05).

### Experimental hyperinsulinemia impairs vasodilation of human skeletal muscle arterioles

**Figure 1:**
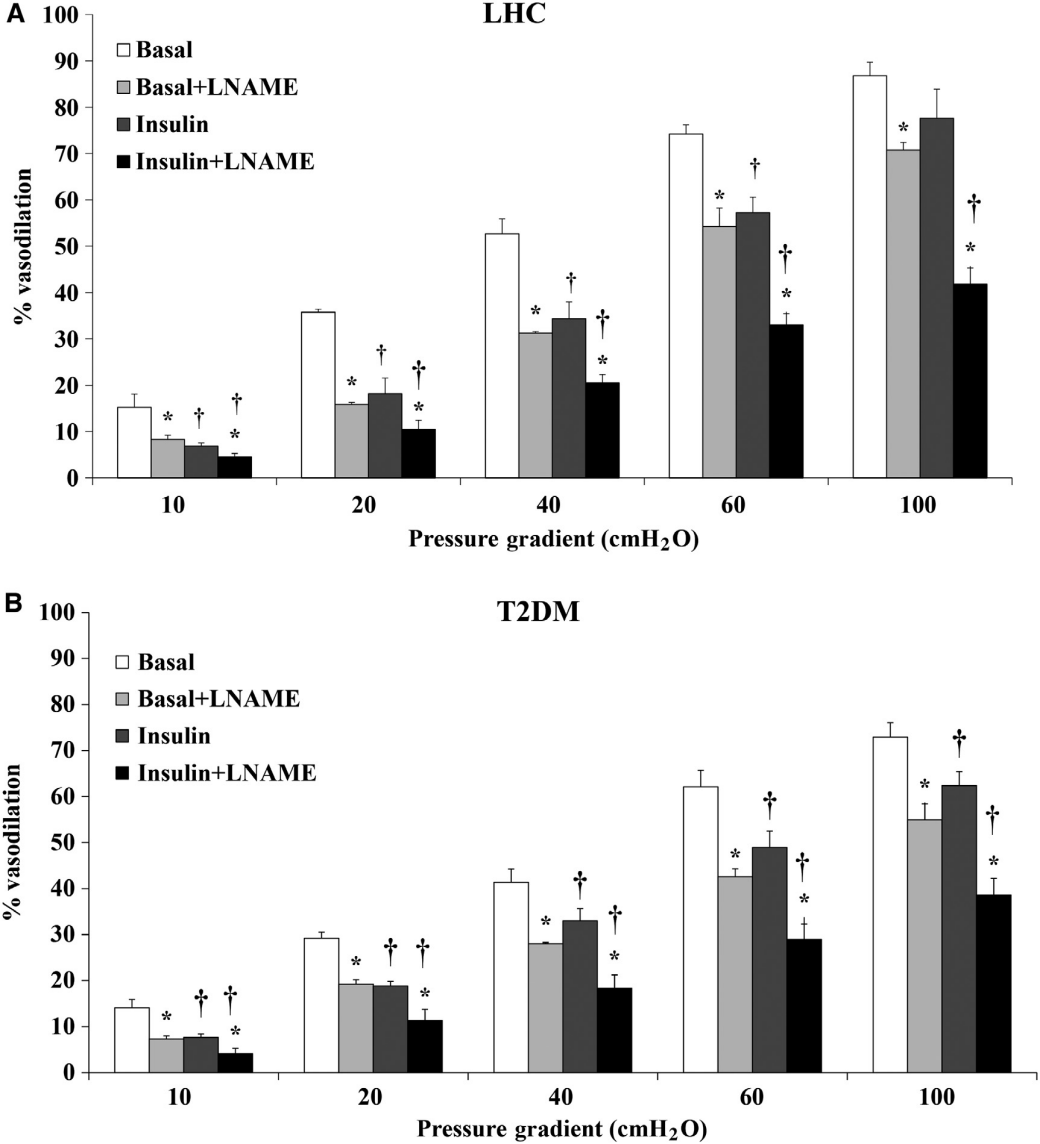
Flow‐induced dilation (FID) is reduced in isolated skeletal muscle arterioles of lean healthy controls (*n* = 5) (A) and T2DM (*n* = 6) (B) at 2 h of hyperinsulinemia. FID was measured during intraluminal flow corresponding to pressure gradients of 10–100 cmH_2_O in the absence or presence of L‐NAME. All values are plotted as means ± SE. *(*P* < 0.05) for L‐NAME and † (*P* < 0.05) for insulin.

**Figure 2:**
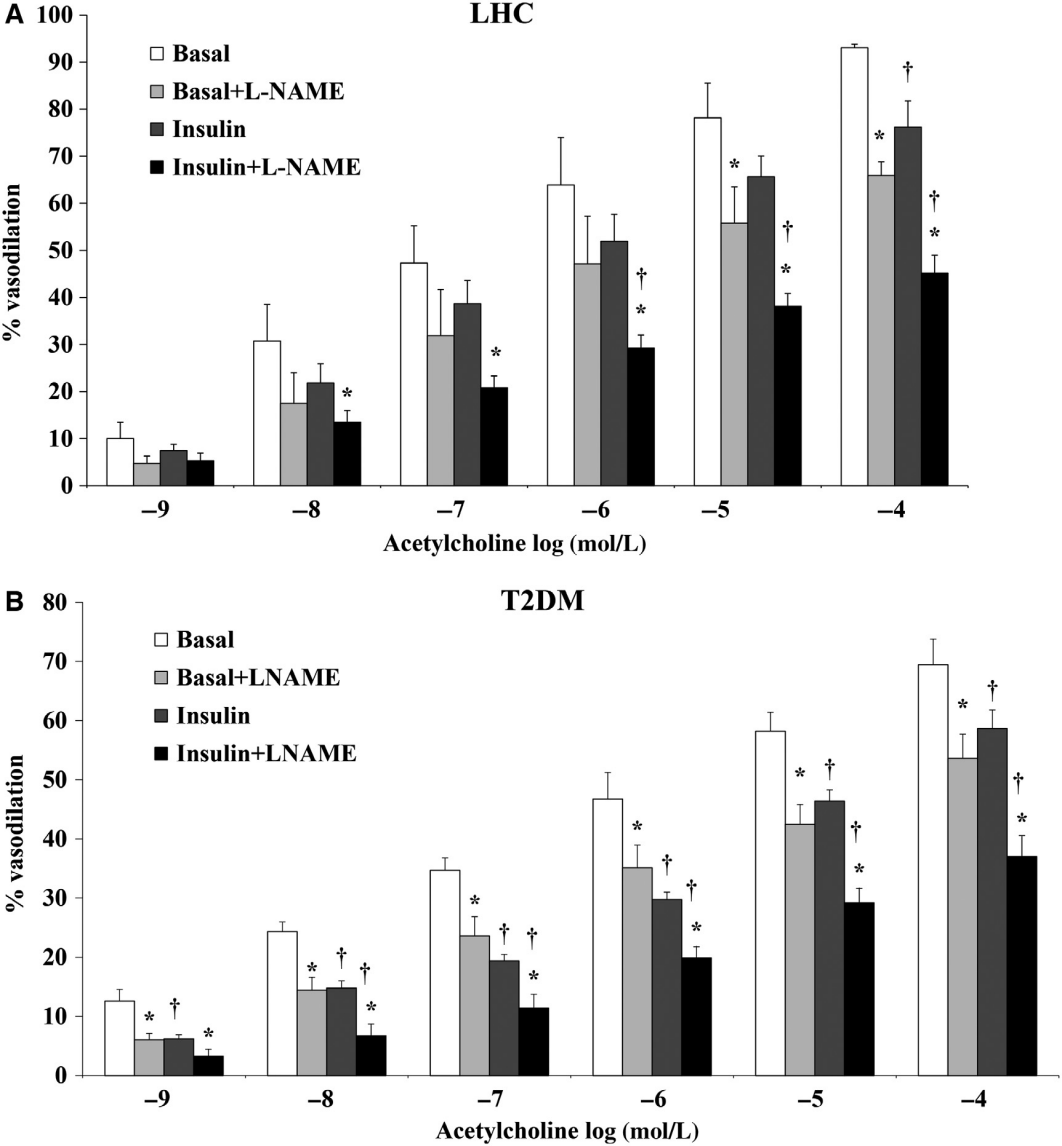
Acetylcholine‐induced dilation is attenuated in isolated skeletal muscle arterioles of lean healthy controls (*n* = 5) (A) and T2DM (*n* = 6) (B) at 2 h of hyperinsulinemia. Arteriolar dilation was measured in response to increased concentrations of acetylcholine (10‐9 to 10‐4 mole/L) in the absence or presence of L‐NAME. All values are plotted as means ± SE. *(*P* < 0.05) for L‐NAME and † (*P* < 0.05) for insulin.

Basal endothelial‐mediated vasodilation of isolated skeletal muscle arterioles was greater in LHCs versus T2DM subjects as determined by FID (*P* = 0.049) and Ach (*P* = 0.046), at all pressure gradients and doses (*P* < 0.001, Figs. 1A, B and 2A, B). Isolated skeletal muscle arterioles demonstrated impaired FID with 2 h of hyperinsulinemia; % of maximum dilation at ∆60 cmH_2_O, which reflects the mean of physiological arteriolar pressure inside the human body, was reduced by 23% in LHCs (*P* = 0.003) (Fig. 1A) and by 22% in subjects with T2DM (*P* = 0.01) (Fig. 1B) relative to basal states. Compared to baseline, FID at ∆60 was also reduced in the presence of L‐NAME (LHCs: −27%, *P* = 0.03; T2DM: −31%, *P* < 0.001). Interestingly, L‐NAME‐induced impairment of FID was greater in insulin‐stimulated arterioles compared to basal (LHCs: −39%, *P* = 0.03; T2DM: −32%, *P* < 0.01), suggesting an increased dependence of FID on NO under hyperinsulinemic conditions. These findings were recapitulated by exposing isolated arterioles to increased concentrations of ACh (Fig. 2A and B), confirming the involvement of endothelium‐dependent mechanisms in the insulin‐induced impairment of vasodilation.

### Hyperinsulinemia reduced circulating NO

**Figure 3:**
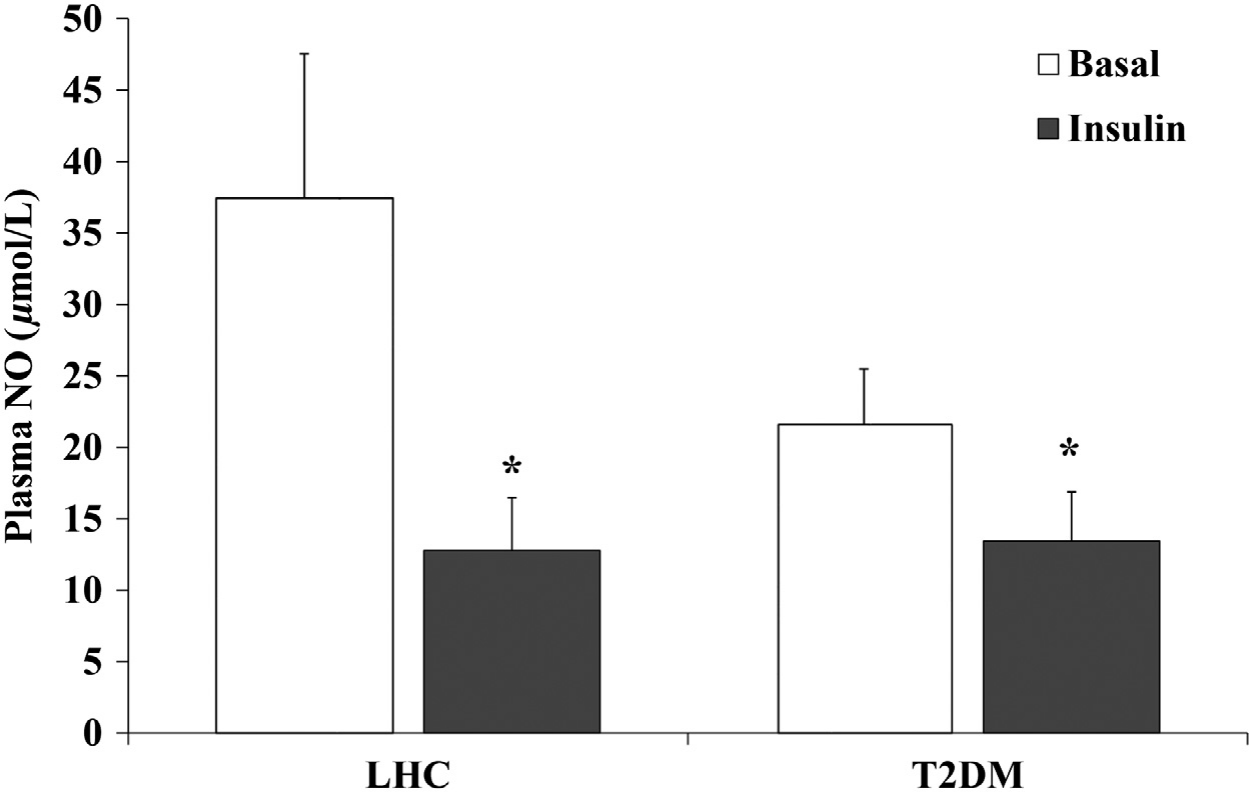
Hyperinsulinemia reduces plasma nitric oxide (NO) (nitrate/nitrite) concentrations in lean healthy controls (*n* = 10) and T2DM (*n* = 9). (A) Plasma nitrite/nitrate concentrations, a marker of NO production, were measured in the basal and hyperinsulinemic states. Results represent the means ± SE for each group. *P < 0.05 for insulin versus basal.

To determine if the observed impairment in FID was accompanied by changes in plasma concentrations of NO, plasma nitrite/nitrate concentrations were measured in LHC and T2DM subjects at basal and insulin‐stimulated conditions. Although not significant, the average plasma NO concentration was higher in LHCs than in T2DM in the basal state (37.4 ± 10.1 *μ*mol/L vs. 21.6 ± 3.9 *μ*mol/L, *P* = 0.08). Plasma NO concentrations during hyperinsulinemia were reduced by 65.9% in LHCs and by 37.8% in T2DM subjects (*P* < 0.01, Fig. 3). Circulating NO was also found to be correlated with FID in the basal state (*R* = 0.7; *P* = 0.04).

### Hyperinsulinemia increases ET‐1 protein expression in skeletal muscle tissue

**Figure 4:**
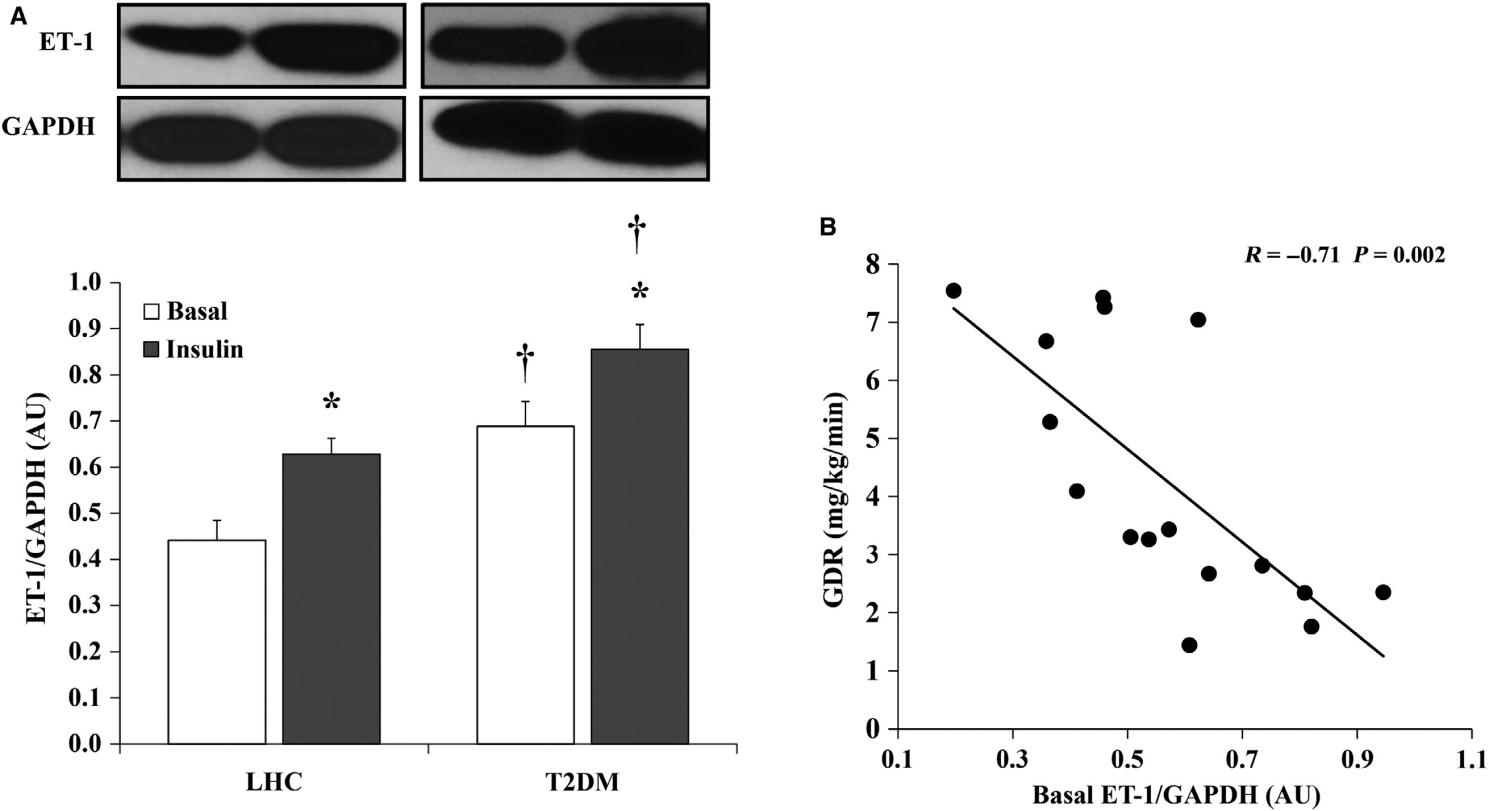
Hyperinsulinemia induces ET‐1 protein expression in skeletal muscle of lean healthy controls (LHCs) (*n* = 8) and T2DM (*n* = 8). (A) Western blot analysis of ET‐1 protein expression in skeletal muscle homogenate. Signal relative intensity was normalized to GAPDH and results represent the means ± SE for each group. *P < 0.05 for insulin versus basal and † (*P* < 0.05) for LHCs versus T2DM. (B) Correlation between ET‐1 protein expression and clamp‐derived glucose disposal rate in the study subjects (*n* = 16).

Skeletal muscle ET‐1 protein expression was greater in T2DM subjects (57%, *P* = 0.003) (Fig. 4A) compared to LHCs. Hyperinsulinemia increased ET‐1 protein expression by 43.2% in LHCs (*P* = 0.007) and by 24.6% in T2DM subjects (*P* = 0.02) compared to basal (Fig. 4A). Similarly, ECE protein, which is responsible for cleaving inactive proendothelin to the active endothelin‐1 peptide, was upregulated in response to hyperinsulinemia in LHCs (basal: 0.80 ± 0.06; insulin: 1.08 ± 0.17, *P* = 0.02); however, no significant changes were detected in T2DM subjects. Basal ET‐1 protein expression was found to be inversely correlated with insulin‐stimulated GDR (*R* = −0.71, *P* = 0.002) (Fig. 4B).

### Hyperinsulinemia increases p‐eNOS (serine 1177) in skeletal muscle of LHCs

**Figure 5:**
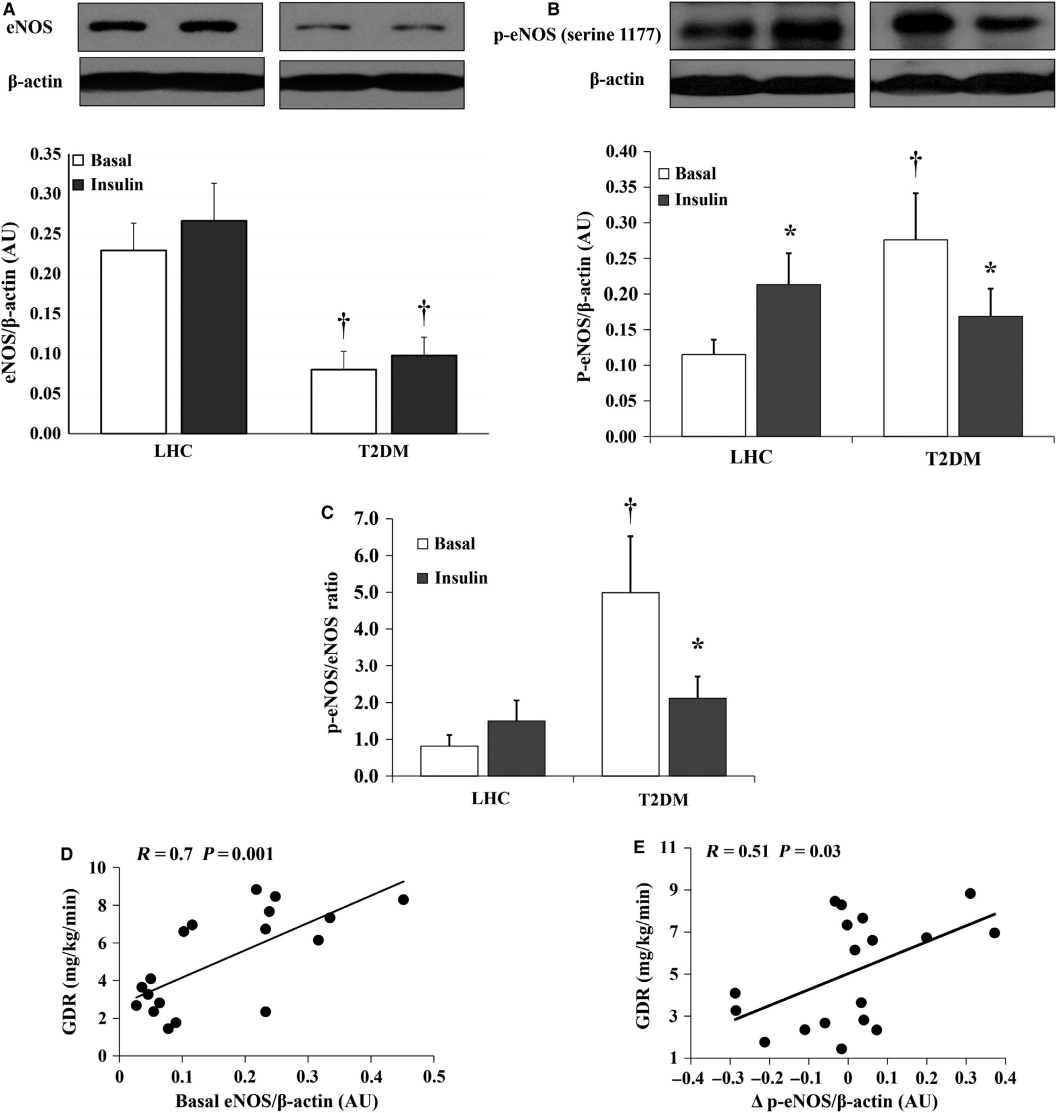
Hyperinsulinemia increases phosphorylated eNOS protein expression in skeletal muscle of lean healthy controls (LHCs) (*n* = 10) but not in T2DM (*n* = 8). Western blot analysis of total eNOS (A) and p‐eNOS (serine 1177) protein expression (B) in skeletal muscle homogenate. Signal relative intensity was normalized to *β*‐actin and results represent the means ± SE for each group. (C) Ratio between the normalized expression of p‐eNOS and eNOS proteins in T2DM subjects and LHCs at 2 h of hyperinsulinemia. Correlation between clamp‐derived glucose disposal rate in study subjects and basal expression of eNOS protein (D) and Δ p‐eNOS (E). *P < 0.05 for insulin versus basal and † (*P* < 0.05) for LHCs versus T2DM.

Figure 5 compares total and phosphorylated eNOS between LHCs and T2DM. Under basal conditions, total eNOS protein was 1.9‐fold higher in LHCs than T2DM subjects (*P* = 0.003), however, the phosphorylated fraction of eNOS (p‐eNOS) was 1.3‐fold greater in the T2DM than the LHCs (*P* = 0.01) (Fig. 5B). Hyperinsulinemia did not induce significant changes in total eNOS protein in LHCs or T2DM subjects (Fig. 5A). In contrast, p‐eNOS showed differential responses to hyperinsulinemia depending on the subject group. In LHCs, p‐eNOS was increased by 75% (*P* = 0.03), whereas in T2DM subjects it was reduced by 39% (*P* = 0.03) (Fig. 5B). Accordingly, p‐eNOS/eNOS ratio was fivefold higher in T2DM (*P* = 0.004) at basal and then reduced by 57.7% during hyperinsulinemia (*P* = 0.02) (Fig. 5C). Significant correlations were observed between GDR and basal expression of eNOS protein (*R* = 0.7, *P* = 0.001) and insulin‐induced changes in p‐eNOS (Δ p‐eNOS) (*R* = 0.51, *P* = 0.03) (Fig. 5D and E, respectively).

### Hyperinsulinemia disturbs the ET‐1/eNOS ratio in T2DM subjects but not in LHCs

**Figure 6:**
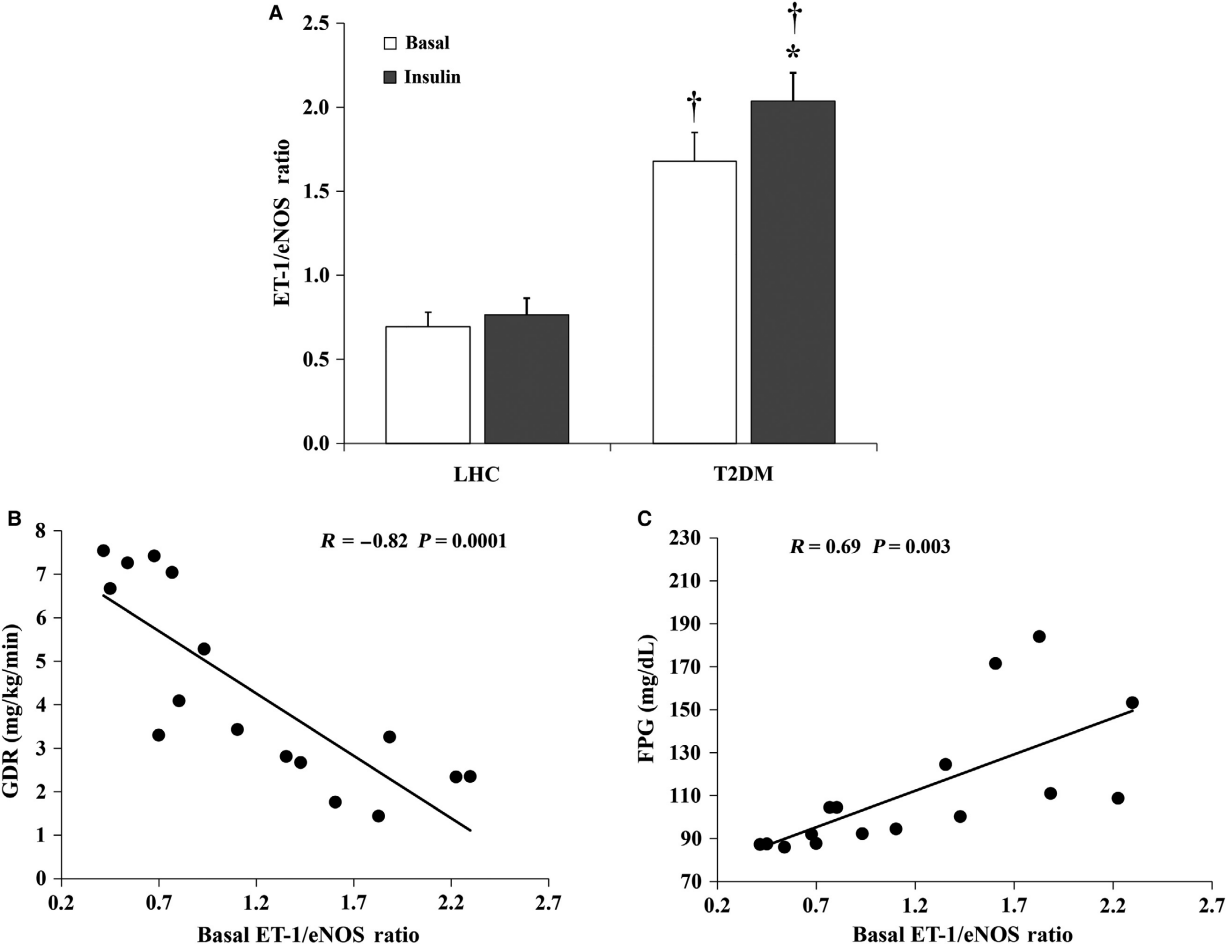
Effects of hyperinsulinemia on the ET‐1/eNOS ratio in skeletal muscle tissue. The ratio between ET‐1 and eNOS (A) in skeletal muscle tissue in T2DM subjects (*n* = 8) and lean healthy controls (LHCs) (*n* = 8). Results represent the means ± SE for each group. *P < 0.05 for insulin versus basal and † (*P* < 0.05) for LHCs versus T2DM. The ratio between the ET‐1 and eNOS protein expression in skeletal muscle tissue of the study subjects (*n* = 16) correlates negatively with glucose disposal rate (B) and positively with FPG (C).

Due to higher ET‐1 and lower eNOS basal protein levels, T2DM subjects displayed greater basal ET‐1/eNOS ratios relative to LHCs (*P* < 0.001) (Fig. 6A). The ET‐1/eNOS ratio was negatively correlated with the insulin‐stimulated GDR (*R* = −0.82, *P* = 0.0001) and positively correlated with fasting plasma glucose (FPG) (*R* = 0.69, *P* = 0.003) (Fig. 6B and C, respectively).

### Hyperinsulinemia modifies ET receptor expression in skeletal muscle of LHCs

**Figure 7:**
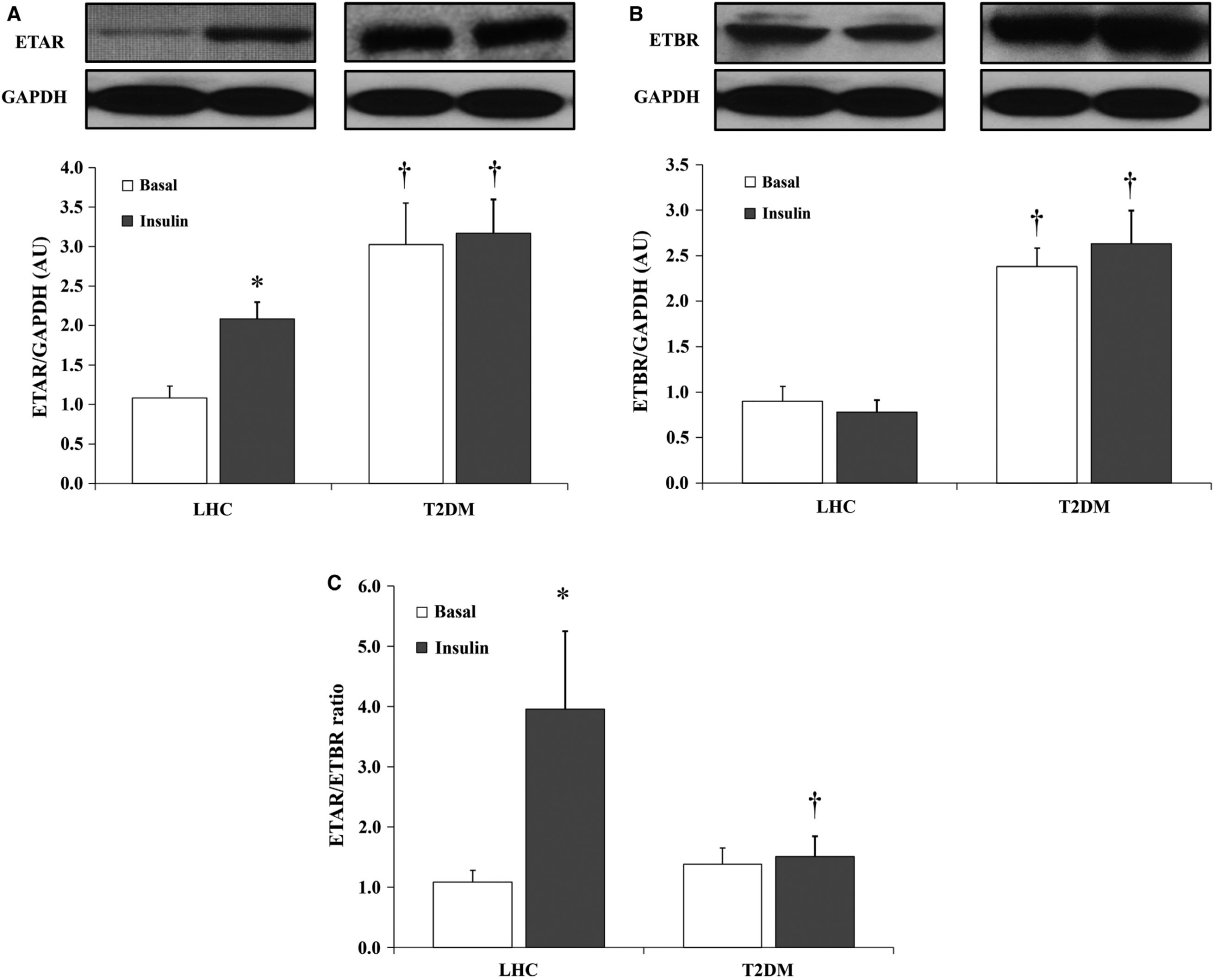
Effects of hyperinsulinemia on ET‐1 receptor protein expression in skeletal muscle of lean healthy controls (LHCs) (*n* = 8) and T2DM (*n* = 8). Western blot analysis ETAR (A) and ETBR (B) protein expression in skeletal muscle homogenate. Signal relative intensity was normalized to GAPDH and results represent the means ± SE for each group. (C) The ratio between normalized expression of ETAR and ETBR proteins in LHCs (*n* = 8) and T2DM (*n* = 8). *P < 0.05 for insulin versus basal and † (*P* < 0.05) for LHCs versus T2DM. *Significant difference between LHC and T2DM (*P* < 0.05).

ET‐1 action is mediated via two types of receptor, ETAR and ETBR. To test the potential downstream effects of hyperinsulinemia‐induced increased ET‐1 protein expression, we measured the protein expression of ETAR and ETBR receptors in LHCs and T2DM subjects at basal and insulin‐stimulated conditions. T2DM subjects had higher basal levels of ETAR (1.8‐fold, *P* = 0.004) and ETBR proteins (1.6‐fold, *P* < 0.0001) relative to LHCs (Fig. 7A and B, respectively). Hyperinsulinemia increased ETAR protein expression in skeletal muscle tissue of LHCs (*P* = 0.004) without any significant effect on ETBR protein expression (Fig. 7A and B). As a result, during hyperinsulinemia, the ETAR/ETBR ratio was increased by 2.7‐fold in LHCs (*P* = 0.03, Fig. 7C). Hyperinsulinemia had no significant effect on either ETAR or ETBR in T2DM subjects.

### Skeletal muscle capillary density is lower in T2DM than LHCs

As expected, the average number of capillaries per square millimeter of surface area (capillary density (CD) and mean capillary to muscle fiber (C/F) ratio were lower in skeletal muscle of T2DM subjects (CD: 3.48 ± 0.17 and C/F ratio: 1.16 ± 0.06) relative to LHCs (CD: 4.85 ± 0.15; C/F ratio: 1.62 ± 0.05, *P* < 0.0001). Although CD and C/F ratios were not found to be correlated with basal plasma NO concentrations (*R* = 0.37, *P* = 0.13), the ratios correlated positively with GDR (*R* = 0.85, *P* < 0.0001) and negatively with body mass index (*R* = −0.75, *P* = 0.0004), FPG (*R* = −0.65, *P* = 0.004), HbA_1c_ (*R* = −0.68, *P* = 0.002), and ETAR protein expression (*R* = −0.81, *P* = 0.01).

## Discussion

The concept of *selective insulin resistance*, in the face of chronic hyperinsulinemia, is now recognized as a major mechanistic factor in the development of endothelial dysfunction and subsequent CVD. The major findings of this work are that acute, experimental hyperinsulinemia impairs endothelial‐mediated FID, reduces plasma NO bioavailability, and disrupts the balance between the vasodilator and the vasoconstrictor pathways in isolated skeletal muscle arterioles in LHCs and T2DM subjects. Furthermore, hyperinsulinemia disturbed the balance between the two main ET‐1 receptors, ETAR and ETBR. Collectively, our data suggest that hyperinsulinemia, independent of impaired insulin action, may contribute to endothelial dysfunction by favoring the predominance of vasoconstrictor signaling. Also, our novel data highlight potential candidate mechanisms for therapuetic target, such as agents that alter the ET‐1 receptor density or ETAR/ETBR ratio. Of clinical relevance, ETAR upregulation is implicated in the pathogenesis of hypertension, endothelial dysfunction, inflammation, and fibrosis (Vignon‐Zellweger et al. 2012). Furthermore, ETAR antagonists are the first‐line treatment option for pulmonary arterial hypertension, and interestingly, are currently being studied for the treatment of diabetic nephropathy (Reichetzeder et al. 2014).

We previously reported that impairments of NO bioavailability and capillary density across the glucose tolerance continuum in obese adults were associated with clamp derived GDRs (Solomon et al. 2011). Consistent with our previous data, we show here that the basal concentrations of plasma NO were considerably lower in the T2DM group than in LHCs. Previous work by Tsukahara et al. (1997) demonstrated increased plasma NO concentrations during experimental hyperinsulinemia in LHCs. In contrast, our data showed a marked decrease in plasma NO in both LHCs and T2DM groups with 2 h of 40 mU/m^2^/min experimental hyperinsulinemia. Interestingly, the magnitude of reduction we observed in plasma NO was greater in the LHC group than the T2DM, which could be explained by the lower basal concentrationsof plasma NO in T2DM subjects. Our data suggest that hyperinsulinemia is capable of reducing circulating NO bioavailability independent of insulin resistance. Despite our observed differences, the use of circulating nitrate and nitrite as a surrogate for NO bioavailability may be limiting due to the contribution of nitrates from the diet. While we made efforts to control dietary intake prior to metabolic testing, we cannot exclude residual dietary effects on our plasma measures. Furthermore, under certain conditions, the nonenzymatic production of NO can be significant and thus we cannot derive the exact proportion of insulin‐mediated NO production from eNOS.

Despite the established vasodilatory action of insulin, human studies show an inconsistent vasomoter response to experimental hyperinsulinemia (Mahmoud et al. 2015). In this study we tried to bridge these gaps by: (1) examining the differential effects of hyperinsulinemia between T2DM subjects and LHCs in order to isolate effects of experimental hyperinsulinemia versus insulin resistance; (2) using an insulin dose (40 mU/m^2^/min) that produces physiological range of peripheral hyperinsulinemia which generally corresponds to peripheral insulin concentrations after a mixed meal (Roden 2007); and (3) employing an innovative ex vivo approach in skeletal muscle that allows direct measurement of arteriolar microvascular diameter under conditions of controlled flow. Using this integrated approach, we demonstrated lower baseline FID and ACh‐induced dilation of isolated skeletal muscle arterioles in subjects with T2DM compared to LHCs. This is consistent with previous studies that reported diminished muscle microvascular perfusion in obese and diabetic individuals using contrast‐enhanced ultrasound (Clerk et al. 2006). With hyperinsulinemia, FID in LHCs was reduced to levels comparable with baseline FID in T2DM subjects, which may indicate a causitive role of hyperinsulinemia in endothelial dysfunction. Interestingly, L‐NAME induced greater reduction in FID in insulin‐stimulated arterioles than arterioles from baseline conditions (Figs. 1 and 2), suggesting an increased dependence of FID on NO bioavailability in the presence of hyperinsulinemia. Previous studies have reported similar findings where larger reductions in blood flow were achieved in response to L‐NAME during hyperinsulinemia than at baseline (Scherrer et al. 1994; Steinberg et al. 1994).

In this study, baseline expression of eNOS phosphorylation in T2DM subjects was unexpectedly higher than LHCs; nevertheless, plasma NO concentrations were lower in T2DM than those in LHCs. Data by Kashyap et al. (2005) reported lower basal NOS activity in T2DM than LHCs assessed by measuring the conversion of l‐arginine into l‐citrulline in skeletal muscle tissue, which is downstream of eNOS phosphorylation. When interpreted in combination with our finding, this might indicate downstream interference with the ability of p‐eNOS to catalyze the conversion of l‐arginine into l‐citrulline and, hence, diminish NO production. This might also promote the assumption that higher basal expression of p‐eNOS in T2DM is a compensatory response to the compromised NO production. However, further work is necessary to confirm this statement and explain this paradoxical finding. Our data also demonstrate a reduction in eNOS phosphorylation in skeletal muscle tissue of T2DM with insulin stimulation which together with the concomitant increase in ET‐1 protein expression may explain the impaired FID in T2DM subjects. Furthermore, in accordance with findings from previous studies (Kashyap et al. 2005; Bradley et al. 2007), LHCs have exhibited consistent increases in eNOS phosphorylation.

Experimental evidence supports the role of insulin in regulating endothelial cell ET‐1 production (Ferri et al. 1995a,b; Chisaki et al. 2003). However, clinical data are contradictory and depend mainly on circulating plasma ET‐1 data that may be of limited value, as ET‐1 predominantly acts in a paracrine manner (Webb 1997). Also, the extent to which insulin‐stimulated ET‐1 production contributes to maintenance of vascular homeostasis in physiological settings versus insulin‐resistant states has not been assessed. Although we did not measure circulating ET‐1 concentrations, we show higher basal protein expression of ECE and ET‐1 in skeletal muscle tissue of T2DM subjects compared with LHCs. These findings are in agreement with previous studies that reported higher circulating concentrations of ET‐1 in obese and T2DM versus healthy individuals (Ferri et al. 1995a,c). It has also been reported that elevated ET‐1 plays a critical role in the development of insulin resistance and endothelial dysfunction in skeletal muscle (Shaw and Boden 2005). Although the insulin‐mediated induction of ET‐1 protein expression in skeletal muscle of T2DM subjects is consistent with others (Ferri et al. 1995c; Rab et al. 2004), our study is the first to demonstrate similar effects in LHCs. This paradoxical similarity in FID and ET‐1 response to insulin between LHCs and T2DM subjects suggests either absence of vascular insulin resistance in T2DM or transient development of vascular insulin resistance in LHCs under conditions of acute hyperinsulinemia. Furthermore, we cannot rule out the possibility that the active form of ET‐1 may not actually be released from skeletal muscle microvessels due to our limitations in detecting the 2 kD active form of ET‐1. However, we are not aware of defects in the processing of pre‐proendothelin‐1 to ET‐1 with insulin stimulation or metabolic disease.

Despite the induction of ET‐1 protein expression, the concomitant stimulation of eNOS phosphorylation led to a preservation of the ratio between these two vasoactive mediators. Thus, investigating the pathway downstream to ET‐1 protein, i.e., ET‐1 action‐mediating receptors, ETAR and ETBR, were essential in this study to detect a deleterious response to hyperinsulinemia that might explain impaired FID in LHCs. Previous work has reported enhanced leg blood flow and skeletal muscle glucose uptake in obese subjects after blockade of ETAR (Lteif et al. 2007). It has been also shown that flow‐mediated dilation with ET‐1 receptor antagonism in obese and T2DM yielded similar data to LHCs (Lteif et al. 2007; Shemyakin et al. 2010), indicating that the basal activity of ET‐1 or the basal expression of ET‐1 receptors may be elevated in subjects with insulin resistance. Furthermore, Shemyakin et al. (2010) demonstrated that dual inhibition of ETAR and ETBR during hyperinsulinemia caused greater improvement of skeletal muscle blood flow and glucose uptake in lean, healthy people than insulin infusion alone. Ross et al. (2007) also showed that whereas ET‐1 infusion alone did not induce significant changes in skeletal muscle hemodynamics or glucose metabolism, concomitant infusion of ET‐1 with insulin reduced blood flow and glucose uptake. These data, along with our current findings, provide evidence that normal levels of insulin maintain a balance between the two opposing ET‐1 receptors and that loss of this balance might be a primary feature in the pathogenesis of hyperinsulinemia‐induced vascular dysfunction even in the absence of underlying insulin resistance.

Given the inherent nature of human investigations, our study is not without limitations in establishing causality and detailed mechanistic insight. Future studies in transgenic animal models or more elaborate human clinical trials of ET‐1 antagonists are needed. Our observations were performed in a small sample size and the data are underpowered for some variables such as the FID measurements due to difficulty in isolating adequate vessels samples. Also, ex vivo measures of FID or reactivity in isolated arterioles removes biological regulation such as neurohumoral activation and metabolic‐induced vasomotor changes (Young et al. 2010). However, this model is a unique aspect of our study that allowed us to observe a microvascular functional consequence to experimental hyperinsulinemia. Furthermore, it was our intent not to match subjects for age or obesity status in an attempt to establish a disease phenotype against a health reference group. We acknowledge that the T2DM disease phenotype is complex and that additional confounding factors that influence vascular function, such as age, obesity, hypertension, hyperglycemia, and inflammation, require both independent and combined study with hyperinsulinemia.

In conclusion, hyperinsulinemia is a hallmark of obesity and T2DM. Mechanistic insight into the role of hyperinsulinemia in skeletal muscle microvascular dysfunction during controlled acute hyperinsulinemia may, therefore, help to uncover treatments for patients exhibiting cardiometabolic risk factors. Our study suggests hyperinsulinemia as an independent and primary factor for the disturbed balance among vasoactive mediators and for the development and advancement of microvascular dysfunction. Moreover, this study serves as a first step toward changing the prevailing concepts about hyperinsulinemia, which may promote reevaluation of the current therapeutic approaches for T2DM such as insulin secretagogues and administration of exogenous insulin.

## Conflicts of Interest

No conflicts of interest were disclosed.
